# A DFT-Based Descriptor to Predict the Water Vapor Corrosion Resistance of Rare-Earth Monosilicates

**DOI:** 10.3390/ma15072414

**Published:** 2022-03-25

**Authors:** Qianqian Wang, Jian Huang

**Affiliations:** 1 State Key Laboratory of High Performance Ceramics and Superfine Microstructure, Shanghai Institute of Ceramics, Chinese Academy of Sciences, Shanghai 201899, China; wangqianqian@student.sic.ac.cn; 2 College of Materials Science and Opto-Electronic Technology, University of Chinese Academy of Sciences, Beijing 100049, China

**Keywords:** ceramics, rare-earth monosilicates, first principles, corrosion

## Abstract

Rare-earth monosilicates are used as environmental barrier coatings (EBCs) due to their excellent water vapor corrosion resistance. However, existing experimental studies on the water vapor corrosion behavior of rare-earth monosilicates are discrepant and even contradictory. Previous theoretical investigations on water vapor corrosion resistance mainly focus on a Mulliken analysis of Si-O bonds in the monosilicates. In this study, the structural and electronic properties of rare-earth monosilicates have been studied by density functional theory (DFT) calculations, and a descriptor correlated to the corrosion resistance has been developed. The maximum isosurface value of the valence band maximum (VBM_Fmax_) can be used to predict the water vapor corrosion resistance of RE_2_SiO_5_. The results show that RE_2_SiO_5_ with a smaller VBM_Fmax_ may have better water vapor corrosion resistance.

## 1. Introduction

Rare-earth (RE) silicates are promising candidates for environmental barrier coatings (EBCs) due to their exceptional high-temperature durability, chemical compatibility and water vapor corrosion resistance [1]. Rare-earth monosilicates, RE_2_SiO_5_, have two types of monoclinic crystalline structures: RE_2_SiO_5_ with a space group of P2_1_/c are called X1 phase when the ionic radius of RE elements (RE = La~Gd) is larger, while the X2 phase with a C_2_/c space group forms by the elements from Tb to Lu [2]. The water vapor corrosion behaviors and mechanisms may be different between the two phases.

Recently, many experimental studies have been carried out regarding the water vapor corrosion resistance of RE_2_SiO_5_ as EBCs. Wang et al. [3] found that X2-RE_2_SiO_5_ are more stable than X1-RE_2_SiO_5_ in high-temperature water steam. Nasiri et al. [4] investigated the water vapor corrosion resistance of RE_2_SiO_5_ (RE = Y, Gd, Er, Yb and Lu) in air with 90% water, and found that their water vapor resistance has the following order: Y_2_SiO_5_ > Er_2_SiO_5_ > Yb_2_SiO_5_ > Lu_2_SiO_5_ > Gd_2_SiO_5_. Klemm et al. [5] studied the water vapor corrosion resistance of Y_2_SiO_5_ and Sc_2_SiO_5_, indicating that Y_2_SiO_5_ exhibited better water vapor corrosion resistance. Unfortunately, the existing experimental studies on the water vapor corrosion behavior of rare-earth monosilicates may be discrepant or even contradictory due to the different experimental conditions [6]. It is worth pointing out that the water vapor corrosion resistance of rare-earth monosilicates can also be evaluated by theoretical calculations. For example, Han et al. [7] studied the water vapor corrosion resistance of X2-RE_2_SiO_5_ (RE = Lu, Yb, Tm, Er, Ho, Dy, Y and Sc) using first-principles calculations. They concluded that the water vapor corrosion resistance of X2-RE_2_SiO_5_ demonstrated the following order: Sc_2_SiO_5_ > Dy_2_SiO_5_ > Y_2_SiO_5_ > Ho_2_SiO_5_ > Er_2_SiO_5_ > Yb_2_SiO_5_ > Tm_2_SiO_5_ > Lu_2_SiO_5_, which shows some disagreement with the experimental findings [3,5,6]. Therefore, it is still a challenge to provide a precise order of the water vapor corrosion resistance of RE_2_SiO_5_.

In previous theoretical studies [7,8], researchers mainly focused on the Si-O bonds instead of RE-O bonds of rare-earth silicates to compare their water vapor corrosion resistances. However, the [REO_x_] polyhedron is less rigid than the [SiO_4_] tetrahedral, and is easier to collapse and react with water molecules. In this work, we used density functional theory (DFT) calculations to investigate the water vapor corrosion resistance of RE_2_SiO_5_ through RE-O bonds. By comparing the structural and electronic properties of RE_2_SiO_5_, we have developed a descriptor, i.e., the maximum isosurface value of the valence band maximum (VBM_Fmax_), to predict their water vapor corrosion resistances.

## 2. Materials and Methods

All DFT calculations were performed using the *Vienna* Ab initio *Simulation Package* (VASP) [9]. The projector augmented-wave (PAW) method and plane-wave basis sets are used [10,11]. The Perdew–Burke–Ernzerhof (PBE) potential is adopted to treat exchange–correlation interactions at the generalized gradient approximation (GGA) level [12]. The cut-off energy for the plane-wave basis was set to 520 eV throughout the present study. The k-point sampling of the Brillouin zone is based on the Monkhorst–Pack method [13]. A 3 × 4 × 4 k-point grid and a 2 × 5 × 3 k-point grid were used for X1-RE_2_SiO_5_ and X2-RE_2_SiO_5_, respectively. The crystal structures were completely optimized by the lattice parameters and internal atomic coordinates until the total energy difference and the forces on atoms were less than 1.0 × 10^−6^ eV and 0.01 eV/Å, respectively. Visualizations of all the structures were performed using VESTA [14].

## 3. Results and Discussion

### 3.1. Crystal Structure of RE_2_SiO_5_

**Figure 1:**
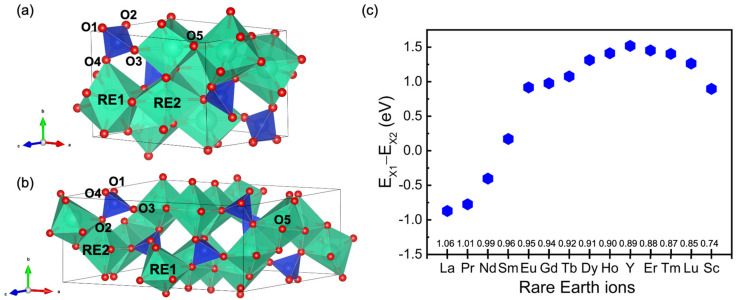
The crystal structure of RE_2_SiO_5_: (**a**) X1-RE_2_SiO_5_ and (**b**) X2-RE_2_SiO_5_. Si tetrahedra, RE polyhedra and O atoms are colored by blue, green and red, respectively. (**c**) The energy difference between the X1 phase and X2 phase for the same RE_2_SiO_5_.

The calculated lattice parameters of RE_2_SiO_5_ and the experimental data are compared in Table 1, and the 1% disagreement suggests that the optimized structures are reasonable. The crystal structures of X1- and X2-RE_2_SiO_5_ are shown in Figure 1a,b. The unit cell of RE_2_SiO_5_ contains 32 atoms, including two different RE sites (labeled as RE1 and RE2), one Si site and five different O sites (labeled as O1–O5). The four O positions of O1–O4 form a Si-centered tetrahedron [SiO_4_], while O5 atoms only loosely bond with rare-earth atoms [2]. The difference between X1-RE_2_SiO_5_ and X2-RE_2_SiO_5_ lies in the coordination number of rare-earth atoms, ranging from nine to seven and seven to six [2]. Figure 1c shows the energy difference between the X1 phase and X2 phase for the same RE_2_SiO_5_. The increasing energy difference indicates that the structural stability of the X1 phase decreases with the increasing ionic radius of RE atoms; RE elements tend to more often form X2-RE_2_SiO_5_ with an increase in the ionic radius of RE atoms [15].

**Figure 2:**
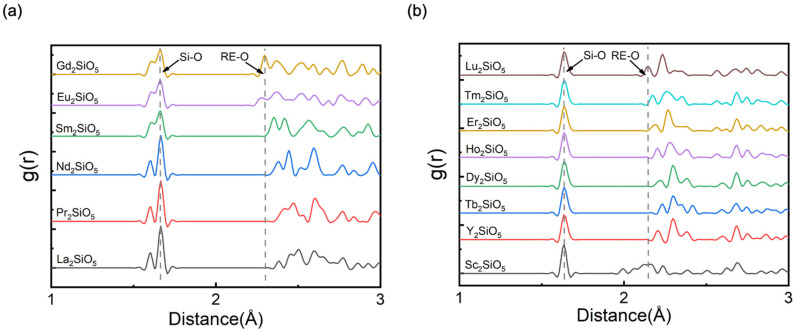
Radial distribution functions, g(r), of Si-O bonds and RE-O bonds in (**a**) X1-RE_2_SiO_5_ and (**b**) X2-RE_2_SiO_5_ compounds.

When exposed to water-vapor-containing environments, RE_2_SiO_5_ will suffer from rapid recession, eventually generating Si(OH)_4_ and RE(OH)_3_ gas [3,6]. Generally, the [REO_x_] polyhedron is less rigid than the [SiO_4_] tetrahedral, and the RE-O bonds tend to show different bonding properties for different rare-earth monosilicates. As shown in Figure 2, we found that the radial distribution functions, g(r), of Si-O bonds remained unchanged, while the distribution of RE-O bonds changed remarkably in both X1- and X2-RE_2_SiO_5_. Therefore, the difference in the water vapor corrosion resistance should be closely related to the changing RE-O bonds and their electronic structures.

### 3.2. The Valence Band Maximum (VBM) of RE_2_SiO_5_

Chemical reactivity can be related to the highest occupied molecular orbital (HOMO) and lowest unoccupied molecular orbital (LUMO) characteristics in molecules, while in bulk materials it can be described by the valence band maximum (VBM) and the conduction band minimum (CBM) [22]. Previously, we [23] investigated the hydration sensitivity of triclinic tricalcium silicate by a combination of DFT calculations and molecular dynamics. We found that the long-term reaction with water molecules is controlled by the proton transport of silicate, and can be intrinsically related to the valence band maximum of the bulk solid. Similarly, for the corrosion behaviors induced by water vapor, the valance band maximum (VBM) of the bulk solid could be used as a descriptor to estimate the water-related corrosion resistance.

**Figure 3:**
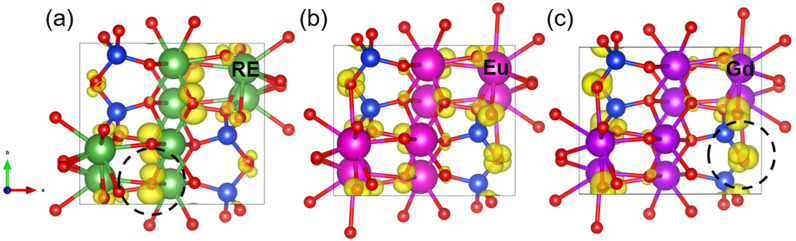
The valence band maximum (VBM) of X1-RE_2_SiO_5_. (**a**) X1-RE_2_SiO_5_ (RE = La, Pr, Nd and Sm). (**b**) Eu_2_SiO_5_. (**c**) Gd_2_SiO_5_. Si atoms (blue) and O atoms (red) are shown. RE atoms (RE = La, Pr, Nd and Sm) are presented by green, Eu is presented by fuchsia and Gd is presented by purple. The isosurface level is set at 0.005 e/Å.

Figure 3 shows the partial charge density at the VBM of X1-RE_2_SiO_5_. The partial charge density at the VBM only exists at the sites of O atoms, suggesting that O atoms would experience an electrophilic attack. Interestingly, the charge densities distributions of X1-RE_2_SiO_5_ are almost unchanged for RE = La, Pr, Nd and Sm, and are mainly located around O5 atoms. However, Gd_2_SiO_5_ shows a completely different profile, where the charge densities are mainly located around O1–O4 atoms. O5 atoms bond with RE much more loosely when compared with the O1–O4 atoms bonding with Si atoms [2]. This may suggest that Gd_2_SiO_5_ is much more stable when reacting with water vapor, indicating that Gd_2_SiO_5_ has better water vapor corrosion resistance. The localization of the valence band maximum in RE_2_SiO_5_ can be related to the water vapor corrosion resistance, and the maximum isosurface value of the valence band maximum (VBM_Fmax_) can be used to describe the electronic localization of RE_2_SiO_5_ [23]. As listed in Table 2, the VBM_Fmax_ of Gd_2_SiO_5_ is much smaller than that of other X1-RE_2_SiO_5_ (RE = La, Pr, Nd, Sm and Eu). More specifically, the VBM_Fmax_ of X1-RE_2_SiO_5_ ranks as Pr_2_SiO_5_ > La_2_SiO_5_ > Nd_2_SiO_5_ > Sm_2_SiO_5_ > Eu_2_SiO_5_ > Gd_2_SiO_5_. Such a decreasing VBM_Fmax_ value corresponds with an increasing water vapor corrosion resistance, implying that Gd_2_SiO_5_ has the best water vapor corrosion resistance among X1-RE_2_SiO_5_.

**Figure 4:**
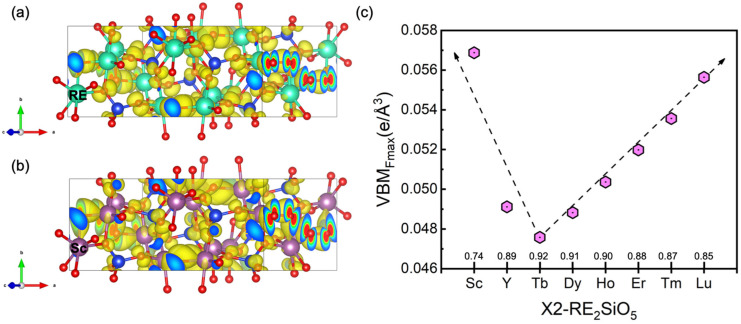
The valence band maximum (VBM) of X2-RE_2_SiO_5_. (**a**) X2-RE_2_SiO_5_ (RE = Tb, Dy, Ho, Er, Tm, Lu and Y). (**b**) Sc_2_SiO_5_. Si atoms (blue) and O atoms (red) are shown. RE atoms (RE = Tb, Dy, Ho, Er, Tm, Lu and Y) are presented by green and Sc is presented by light purple. The isosurface level is set at 0.001e/Å. (**c**) The maximum isosurface value of the valence band maximum (VBM_Fmax_) of X2-RE_2_SiO_5_ (RE = Tb, Dy, Ho, Er, Tm, Lu, Sc and Y).

For the X2-RE_2_SiO_5_ (RE = Tb, Dy, Ho, Er, Tm, Lu, Sc and Y) compounds, their VBM_Fmax_ is also analyzed, as shown in Figure 4. It is worth pointing out that the charge densities distributions of X2-RE_2_SiO_5_ are almost unchanged for RE = Tb, Dy, Ho, Er, Tm, Lu and Y. However, Sc_2_SiO_5_ shows a slightly different profile, where the charge densities around O atoms are a little more than those of other X2-RE_2_SiO_5_. This could imply that Sc_2_SiO_5_ is less stable when reacting with water vapor, indicating that Sc_2_SiO_5_ has worse water vapor corrosion resistance. Both Y_2_SiO_5_ and Er_2_SiO_5_ have a smaller value of the VBM_Fmax_ than that of Gd_2_SiO_5_, indicating that Gd_2_SiO_5_ has worse water vapor corrosion resistance. This is in agreement with the experimental outcomes of Wang et al. [3]. On the basis of the trend in Figure 3c, Y_2_SiO_5_ has better water vapor corrosion resistance than Sc_2_SiO_5_, which is consistent with what Klemm et al. concluded [5]. The decreasing order of RE_2_SiO_5_ (RE = Y, Er and Lu) in our results may also provide an explanation for the experimental results conducted by Nasiri et al. [4]. Additionally, we can conclude that the water vapor corrosion resistance of X2-RE_2_SiO_5_ has the following order: Tb_2_SiO_5_ > Dy_2_SiO_5_ > Y_2_SiO_5_ > Ho_2_SiO_5_ > Er_2_SiO_5_ > Tm_2_SiO_5_ > Lu_2_SiO_5_ > Sc_2_SiO_5_.

## 4. Conclusions

The water vapor corrosion resistance of RE_2_SiO_5_ (RE = La, Pr, Nd, Sm, Eu, Gd, Tb, Dy, Ho, Er, Tm, Lu, Sc and Y) was studied based on DFT calculations. A DFT-based descriptor, the maximum isosurface value of the valence band maximum (VBM_Fmax_), was developed to predict the corrosion resistance for both X1- and X2-RE_2_SiO_5_. According to the proposed descriptor, it was found that Gd_2_SiO_5_ had the best water vapor corrosion resistance in X1-RE_2_SiO_5_ and that the water vapor corrosion resistance of X2-RE_2_SiO_5_ has the following order: Tb_2_SiO_5_ > Dy_2_SiO_5_ > Y_2_SiO_5_ > Ho_2_SiO_5_ > Er_2_SiO_5_ > Tm_2_SiO_5_ > Lu_2_SiO_5_ > Sc_2_SiO_5_.

## Figures and Tables

**Table 1:** Experimental and calculated lattice parameters of X1- and X2-RE_2_SiO_5_.

	Method	a (Å)	b (Å)	c (Å)	β (°)	Volume (Å^3^)
La2SiO5	Expt. [16] Calc.	9.3320 9.3564	7.5088 7.7155	7.0332 7.0168	108.6790 109.0060	466.8700 478.9250
Pr2SiO5	Expt. [17] Calc.	9.2530 9.3420	7.3010 7.5586	6.9340 6.9548	108.1500 108.7720	445.1000 464.9780
Nd2SiO5	Expt. [18] Calc.	9.2295 9.3039	7.2848 7.4606	6.8744 6.9006	108.1990 108.5470	439.0800 454.1070
Sm2SiO5	Expt. [17] Calc.	9.1610 9.2362	7.1120 7.2989	6.8210 6.8096	107.5100 108.1510	424.4000 436.2150
Eu2SiO5	Expt. [17] Calc.	9.1420 9.1706	7.0540 7.2362	6.7900 6.7107	107.5300 107.7030	417.9000 424.2390
Gd2SiO5	Expt. [19] Calc.	9.1200 9.1758	7.0600 7.0566	6.7300 6.7732	107.5800 107.2030	413.0900 419.0640
Tb2SiO5	Expt. Calc.	14.3660 14.5834	6.6976 6.8319	10.3633 10.5585	122.2190 122.1380	843.5900 890.7910
Dy2SiO5	Expt. [17] Calc.	14.3800 14.5296	6.7400 6.8106	10.4200 10.5085	122.0000 122.1140	856.5000 880.7640
Ho2SiO5	Expt. [17] Calc.	14.3500 14.4802	6.7100 6.7785	10.3700 10.4563	122.2000 122.0950	843.0000 869.4740
Er2SiO5	Expt. [20] Calc.	14.3660 14.4344	6.6976 6.7503	10.3633 10.4101	122.2190 122.1120	843.5900 859.1420
Tm2SiO5	Expt. [17] Calc.	14.3020 14.3815	6.6620 6.7197	10.3130 10.3633	122.2100 122.0910	828.5000 848.4790
Lu2SiO5	Expt. [21] Calc.	14.2774 14.2753	6.6398 6.6687	10.2465 10.2827	122.2240 121.9780	821.7400 830.3420
Y2SiO5	Expt. Calc.	14.5643 14.5111	6.8354 6.8113	10.5570 10.5122	122.1320 122.0870	889.9930 880.3030
Sc2SiO5	Expt. Calc.	13.8636 13.7566	6.4838 6.4896	9.9120 10.0833	121.5360 120.8350	759.3900 772.9450

**Table 2:** The maximum isosurface value of the valence band maximum (VBM_Fmax_) of X1-RE_2_SiO_5_.

	La_2_SiO_5_	Pr_2_SiO_5_	Nd_2_SiO_5_	Sm_2_SiO_5_	Eu_2_SiO_5_	Gd_2_SiO_5_
VBM_Fmax_(e/Å^3^)	0.098	0.099	0.097	0.094	0.060	0.053

## Data Availability

The data presented in this study are available on request from the corresponding author.

## References

[1] Lee K.N., Fox D.S., Bansal N.P. (2005). Rare earth silicate environmental barrier coatings for SiC/SiC composites and Si3N4 ceramics. J. Eur. Ceram. Soc..

[2] Tian Z.L., Wang J.Y. (2018). Research Progress of Rare Earth Silicate Ceramics. J. Adv. Ceram..

[3] Wang Y., Niu Y., Zhong X., Shi M., Mao F., Zhang L., Li Q., Zheng X. (2020). Water vapor corrosion behaviors of plasma sprayed RE2SiO5 (RE = Gd, Y, Er) coatings. Corros. Sci..

[4] Al Nasiri N., Patra N., Jayaseelan D.D., Lee W.E. (2017). Water vapour corrosion of rare earth monosilicates for environmental barrier coating application. Ceram. Int..

[5] Klemm H. (2010). Silicon Nitride for High-Temperature Applications. J. Am. Ceram. Soc..

[6] Tian Z., Zhang J., Sun L., Zheng L., Wang J. (2019). Robust hydrophobicity and evaporation inertness of rare-earth monosilicates in hot steam at very high temperature. J. Am. Ceram. Soc..

[7] Han J., Wang Y., Liu R., Jiang D. (2018). Study on water vapor corrosion resistance of rare earth monosilicates RE2SiO5 (RE = Lu, Yb, Tm, Er, Ho, Dy, Y, and Sc) from first-principles calculations. Heliyon.

[8] Wang Y., Liu J. (2009). First-principles investigation on the corrosion resistance of rare earth disilicates in water vapor. J. Eur. Ceram. Soc..

[9] Kresse J.F.L.G. (1996). Efficient iterative schemes for ab initio total-energy calculations using a plane-wave basis set. Phys. Rev. B.

[10] Blochl P.E. (1994). Projector augmented-wave method. Phys. Rev. B.

[11] Blochl P.E., Jepsen O., Andersen O.K. (1994). Improved tetrahedron method for Brillouin-zone integrations. Phys. Rev. B.

[12] Perdew J.P., Burke K., Ernzerhof M. (1996). Generalized Gradient Approximation Made Simple. Phys. Rev. Lett..

[13] Monkhorst H.J., Pack J.D. (1976). Special points for Brillouin-zone integrations. Phys. Rev. B.

[14] Momma K., Izumi F. (2011). VESTA 3 for three-dimensional visualization of crystal, volumetric and morphology data. J. Appl. Crystallogr..

[15] Wang J.G., Tian S.J., Li G.B., Liao F.H., Jing X.P. (2001). Preparation and X-ray characterization of low-temperature phases of R_2_SiO_5_ (R = rare earth elements). Mater. Res. Bull..

[16] Fukuda K., Iwata T., Champion E. (2006). Crystal structure of lanthanum oxyorthosilicate, La_2_SiO_5_. Powder Diffr..

[17] Felsche J. (1973). The crystal chemistry of the rare-earth silicates. Struct. Bond..

[18] León-Reina L., Porras-Vázquez J.M., Losilla E.R., Moreno-Real L., Aranda M.A.G. (2008). Structure and oxide anion conductivity in Ln_2_(TO_4_)O (Ln=La, Nd; T=Ge, Si). J Solid State Chem..

[19] Smolin Y.I., Tkachev S.P. (1969). Determination of the structure of gadolinium oxyorthosilicate (Gd2O3)(SiO2). Kristallografiya.

[20] Phanon D., Černý R. (2008). Crystal Structure of the B-type Dierbium Oxideortho-Oxosilicate Er2O[SiO4]. Z. Anorg. Und Allg. Chem..

[21] Gustafsson T., Kliintenberg M., Derenzo S.E., Weber M.J., Thomas J.O. (2001). Structure of Lu2 Si O5 using single-crystal X-ray and neutron diffraction. Acta Crystallogr. C.

[22] DeKock R.L., Barbachyn M.R. (1979). Proton Affinity, Ionization Energy, and the Nature of Frontier Orbital Electron Density. J. Am. Chem. Soc..

[23] Huang J., Wang B., Yu Y., Valenzano L., Bauchy M., Sant G. (2015). Electronic Origin of Doping-Induced Enhancements of Reactivity: Case Study of Tricalcium Silicate. J. Phys. Chem. C.

